# General Surgery Faculty Knowledge and Perceptions of Breast Pumping Amongst Postpartum Surgical Residents

**DOI:** 10.1007/s00268-023-07005-5

**Published:** 2023-04-27

**Authors:** Devon C. Freudenberger, Kelly M. Herremans, Andrea N. Riner, Vignesh Vudatha, Kandace P. McGuire, Rahul J. Anand, Jose G. Trevino

**Affiliations:** 1 Department of Surgery, Virginia Commonwealth University School of Medicine, 1200 E. Broad St., PO Box 980011, Richmond, VA 23219 USA; 2 Department of Surgery, University of Florida College of Medicine, 1600 SW Archer Rd., Gainesville, FL 32608 USA

## Abstract

**Background:**

There is a lack of data regarding the knowledge and perceptions teaching faculty possess about breast pumping among general surgery residents despite breast pumping becoming more common during training. This study aimed to examine faculty knowledge and perceptions of breast pumping amongst general surgery residents.

**Methods:**

A 29-question survey measuring knowledge and perceptions about breast pumping was administered online to United States teaching faculty from March–April 2022. Descriptive statistics were used to characterize responses, Fisher’s exact test was used to report differences in responses by surgeon sex and age, and qualitative analysis identified recurrent themes.

**Results:**

156 responses were analyzed; 58.6% were male and 41.4% were female, and the majority (63.5%) were less than 50 years old. Nearly all (97.7%) women with children breast pumped, while 75.3% of men with children had partners who pumped. Men more often than women indicated “I don’t know” when asked about frequency (24.7 vs. 7.9%, *p* = 0.041) and duration (25.0 vs. 9.5%, *p* = 0.007) of pumping. Nearly all surgeons are comfortable (97.4%) discussing lactation needs and support (98.1%) breast pumping, yet only two-thirds feel their institutions are supportive. Almost half (41.0%) of surgeons agreed that breast pumping does not impact operating room workflow. Recurring themes included normalizing breast pumping, creating change to better support residents, and communicating needs between all parties.

**Conclusions:**

Teaching faculty may have supportive perceptions about breast pumping, but knowledge gaps may hinder greater levels of support. Opportunities exist for increased faculty education, communication, and policies to better support breast pumping residents.

**Supplementary Information:**

The online version contains supplementary material available at 10.1007/s00268-023-07005-5.

## Introduction

Breast milk is the best source of nutrition for newborns and infants [1]. The American Academy of Pediatrics recommends its exclusive use through the first six months of life. Recently, updated guidelines recommended breastfeeding for two years and beyond as desired by mother and child [2]. Despite guidance, breastfeeding rates drop from 83.9 to only 56.7% six months after birth [3]. Though the circumstances and decision to stop breastfeeding is unique to everyone, returning to work undoubtedly contributes. Breastfeeding consumes ~ 25% of the body’s daily energy and requires ~ 500 h over six months [4, 5]. Returning to work while balancing these added demands requires the availability of appropriate facilities and allocated time to meet breastfeeding/pumping needs [6, 7]. Further, organizational and co-worker support are essential for continuing breastfeeding once returning to work [8–12].

Women physicians face additional challenges once returning to work, including limited maternity leave, long work hours, lack of lactation facilities, lack of policies/support, limited flexibility in schedules, and increased work-related stressors that may negatively impact milk production [7, 13–18]. Unfortunately, women physicians also experience stigma and discrimination when breastfeeding/pumping [15, 16, 19, 20]. These obstacles are encountered throughout training and in all fields: surgical residency is not immune [21, 22]. As the number of female trainees in surgery increases, childbearing during training and practice has become omnipresent, including the practice of breast pumping at work [23–26]. However, in a 2018 study, despite 95.6% of general surgery residents indicating breastfeeding was important to them, 58.1% stopped early due to challenges in the workplace [27].

Gender discrimination among female general surgery residents in the United States is common with ~ 80% of female surgery trainees experiencing it [28]. To help alleviate this pervasive issue, it is critical to identify areas in training where this may be occurring. One area may be the support provided to residents who breastfeed/pump. To our knowledge there is a paucity of data on the knowledge and perceptions that teaching faculty have about breast pumping in surgical trainees. In this study we sought to (1) evaluate the knowledge and perceptions of general surgery teaching faculty toward breast pumping residents and (2) identify areas of improvement. We hypothesized that a general lack of knowledge about breast pumping may ultimately contribute to misperceptions about its practice.

## Material and methods

### Survey development

A 29-question survey was developed to evaluate knowledge and perceptions of breast pumping. Questions were generated from interviews of general surgery residents and faculty, noting key themes. The survey was reviewed by residents, faculty, and survey-development experts. After piloting it at two academic general surgery programs, a final version (Supplemental Material) was created using QuestionPro Survey Software (Survey Analytics LLC, Austin, TX, USA) and approved exempt by the Virginia Commonwealth University institutional review board (IRB#: HM20023438).

### Survey content

After consenting, teaching faculty were permitted to complete the survey. Self-reporting of sex, age, specialty, and practice was included. Surgeons were asked if they had biological children. If “yes,” they were then asked if they personally breast pumped or their partner breast pumped.

Questions then examined knowledge of breast pumping such as duration, frequency, and location of lactation facilities. A 5-point Likert scale of *Strongly Disagree* to *Strongly Agree* assessed surgeon perceptions to how breast pumping impacts the daily workflow, and their personal and institutional support for breast pumping. Finally, an open-ended question provided opportunity for any remaining comments.

### Survey distribution

To target teaching faculty, the Association of Program Directors in Surgery email listserv was selected and after approval, the survey was distributed three times with a request to forward the survey to teaching faculty [29]. Participation was voluntary, anonymous, singular, and without compensation. Data were collected over four weeks from March–April 2022.

### Statistical analysis

According to the American Association of Medical Colleges there are approximately 6,300 academic general surgery and subspecialty-related surgeons in the United States [30]. Using a power of 80% with a margin of error of 5%, a sample size of 160 responses was calculated. To address missingness, responses that contained 20% or more missing items were excluded from analysis.

Responses to Likert-scale questions were converted to “Agreed,” “Neutral,” and “Disagreed.” During the survey development process, two specific demographic subgroups were identified as having potentially differing perceptions: surgeon sex and age. Descriptive statistics and Fisher’s exact test were used to analyze differences in subgroup responses (male vs. female and < 50 years old vs. ≥ 50 years old). All statistical analyses were completed using IBM SPSS Statistics for Windows, Version 28.0.1.0 (IBM Corp., Armonk, NY, USA), with an alpha value of 0.05.

### Thematic analysis

Recurring themes were identified from open-ended responses. Two authors (D.C.F. and V.V.) independently reviewed all responses and generated codes. Codes were used to identify recurring themes on multiple iterations of review. Codebooks were reconciled between authors until full agreement and generated using Dedoose Version 9.0.46 (SocioCultural Research Consultants, LLC, Los Angeles, CA, USA).

Of note, throughout this manuscript we use the terms “female” or “women,” but understand that some lactating individuals may identify with a different gender identity.

## Results

**Fig. 1:**
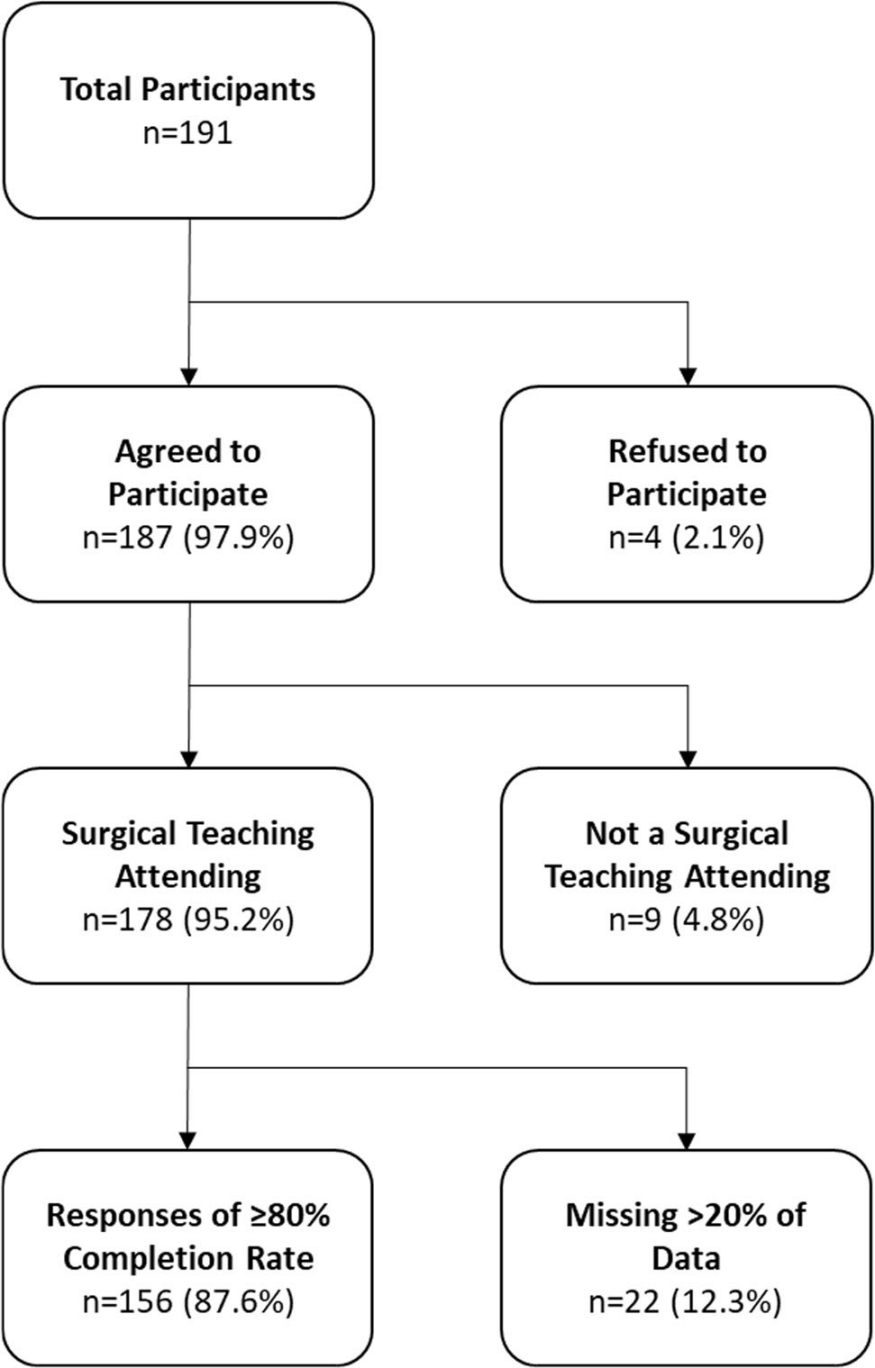
Survey response breakdown for 156 responses included in the final data analysis

In total, 187 of 191 (97.9%) surgeons consented to participate. Of these, 178 (95.2%) respondents identified as teaching faculty and could complete the remainder of the survey. A total of 156 (87.6%) responses had ≥ 80% complete data and were analyzed (Fig. 1). It is unknown how many faculty were reached with this survey, so a formal response rate cannot be determined.

### Participant demographics

Responses were collected from 89 male surgeons (58.6%) and 63 female surgeons (41.4%) (Table 1). Most surgeons were < 50 years of age (63.5%, 99/156) and have practiced for < 10 years (53.2%, 83/156). The top five specialties were acute care/trauma/critical care (32.9%, 51/155), general surgery (23.9%, 37/155), surgical oncology (7.1%, 11/155), colorectal (7.1%, 11/155), and pediatric surgery (5.8%, 9/155). Responses were collected throughout the United States with most surgeons practicing in urban (75.6%, 118/156) and academic (73.7%, 115/156) settings. Over forty percent (42.3%, 66/156) of surgeons are involved in their residency’s leadership.

**Table 1:** Survey respondent demographics and surgical practice characteristics (n = 156)

Characteristic	No. (%)
*Sex*	
Male	89 (58.6)
Female	63 (41.4)
*Age*	
30–39 years old	44 (28.2)
40–49 years old	55 (35.3)
50–59 years old	32 (20.5)
60–69 years old	20 (12.8)
≥ 70 years old	5 (3.2)
*Race*	
White/Caucasian	135 (86.5)
Black/African American	2 (1.3)
American Indian/Alaska Native	0 (0.0)
Asian	13 (8.3)
Native Hawaiian	0 (0.0)
Other Pacific Islander	0 (0.0)
Other	6 (3.8)
*Ethnicity*	
Non-Hispanic	143 (92.3)
Hispanic	12 (7.7)
*Years practicing as an attending*	
0–5 years	50 (32.1)
6–10 years	33 (21.2)
11–15 years	22 (14.1)
16–20 years	11 (7.1)
21–25 years	13 (8.3)
> 25 years	27 (17.3)
*Surgical specialty*	
Acute Care/Trauma/Critical Care	51 (32.9)
Bariatric/Metabolic	8 (5.2)
Breast	6 (3.9)
Burn	0 (0.0)
Cardiothoracic	7 (4.5)
Colorectal	11 (7.1)
Endocrine	3 (1.9)
General	37 (23.9)
Hepatopancreaticobiliary	2 (1.3)
Pediatric	9 (5.8)
Plastics	1 (0.6)
Surgical oncology	11 (7.1)
Transplant	4 (2.6)
Vascular	5 (3.2)
*Location*	
Northeast	20 (12.8)
Southeast	53 (34.0)
Midwest	50 (32.1)
Southwest	15 (9.6)
West	18 (11.5)
*Location of practice*	
Rural	11 (7.1)
Urban	118 (75.6)
Suburban	27 (17.3)
*Type of hospital*	
Academic	115 (73.7)
Community	12 (7.7)
Hybrid	26 (16.7)
Federal	2 (1.3)
Other	1 (0.6)
Involvement in program leadership	66 (42.3)
Have biologic children	119 (76.3)
Personal experience with pumping (Females)	43 (97.7)
Partner experience with pumping (Males)	55 (75.3)
Overall experience with pumping	98 (63.2)

Three quarters (76.3%, 119/156) of respondents have children. Of the 44 female surgeons with children, 43 (97.7%) stated that they breast pumped, while 75.3% (55/73) of male surgeons with children indicated that their partner breast pumped.

### Breast pumping knowledge

Three questions evaluated teaching faculty knowledge of breast pumping (Table 2). When asked how long it may take a resident to breast pump, 23.7% (37/156) responded 16–30 min, 39.7% (62/156) responded 31–45 min, and 18.6% (29/156) responded “I don’t know.” Responses varied significantly by surgeon sex with male surgeons more often indicating “I don’t know” than female surgeons (24.7 [22/89] vs. 7.9% [5/63], *p* = 0.041). When asked how often someone may need to breast pump during the day, almost two–thirds (64.5%, 100/155]) of surgeons chose “every 3–4 h” and 19.4% (30/155) indicated they do not know. Responses varied significantly by surgeon sex and age with male surgeons and those ≥ 50 years old choosing “I don’t know” more often than female surgeons (25.0 [22/88] vs. 9.5% [6/63], *p* = 0.007) and those < 50 years old (31.6 [18/57] vs. 12.2% [12/98], *p* = 0.001), respectively. The location of lactation facilities was not uniform. Only 12.3% (19/155) of respondents stated there are lactation facilities adjacent to the operating rooms. A substantial proportion (29.7%, 46/155) did not know where facilities exist with men more likely than women indicating this (39.8 [35/88] vs. 15.9% [10/63], *p* = 0.016).

**Table 2:** Summary of responses to non-Likert questions (n = 156)

Question	No. (%)
The nearest OR accessible lactation facilities are found…	
Adjacent to the ORS	19 (12.3)
On the same floor as the ORs	31 (20.0)
In the same building, but different floor than the ORs	51 (32.9)
In a different building than the ORs	3 (1.9)
There are no lactation facilities	5 (3.2)
I don't know	46 (29.7)
How long does it take to breast pump?	
0–15 min	2 (1.3)
16–30 min	37 (23.7)
31–45 min	62 (39.7)
46–60 min	20 (12.8)
> 1 h	6 (3.8)
I don't know	29 (18.6)
How often does someone need to breast pump a day?	
Every 1–2 h	2 (1.3)
Every 3–4 h	100 (64.5)
Every 5–6 h	21 (13.5)
Every 7–8 h	2 (1.3)
I don't know	30 (19.4)

### Breast pumping perceptions

**Fig. 2:**
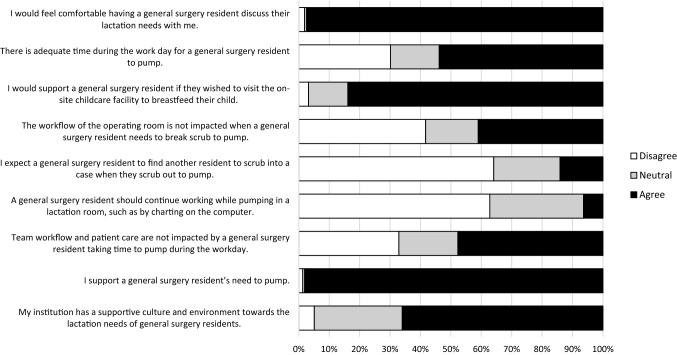
Summary of percentage of support or lack of support for all Likert-scale questions

Responses to breast pumping perception questions are summarized in Fig. 2. Nearly all respondents (97.4%, 152/156) agreed they are comfortable discussing lactation/breast pumping needs with a resident and 98.1% (153/156) support a resident’s need to breast pump. Despite this support, only two–thirds (66.0%, 103/156) feel that their institutions have a supportive culture for breast pumping. Female compared to male (44.4 [28/63] vs 20.2% [18/89], *p* = 0.006) and younger compared to older (41.4 [41/99] vs. 10.5% [6/57], *p* < 0.001) surgeons disagreed that there is adequate time to pump during the workday. With respect to workflow, only 41.0% (64/156) and 47.7% (74/155) of surgeons do not believe a resident excusing themselves to breast pump negatively affects workflow in the operating room or floors, respectively. Furthermore, 14.1% (22/156) of surgeons expected a resident to find a replacement during a case if they scrub out to breast pump. Additionally, 6.4% (10/156) of all respondents believe that residents should continue working while pumping (e.g., charting on the computer); these beliefs did not vary by surgeon sex or age.

### Thematic results

Of the 156 responses, 47 (30.1%) surgeons responded to the open–ended question with most responses from women (62.2%, n = 28). Four themes were identified and are presented with representative quotes (Table 3).

**Table 3:** Recurring themes with representative quotes identified from thematic analysis

*Theme 1: Breast pumping is a natural activity that should be normalized* “Faculty should not treat breast pumping residents like they are doing something taboo…breast pumping/ breast feeding has been done by millions of women, across the world, throughout the history of mankind- I think we just need to work on normalizing this and bringing awareness to it.” “It should not be considered a luxury or a 'break' to pump— this is a necessary function of a working mother in the postpartum year(s), both to feed her baby and to prevent issues such as clogged ducts and mastitis. Some people can pump while they are working and get other things done in this time, but not everyone has that benefit, and many people need a longer, hands-on time to pump. Advocating for this is important to make parents returning to work feel welcomed back; the transition is hard enough as it is.” “Pumping needs to be normalized-we need more awareness around the amount of time it requires, especially if there isn't a lactation room that is readily available. Most people (unless they've experienced pumping) have NO IDEA how difficult this can be, especially as a surgery resident.”
*Theme 2: There is need for changes to better support breast pumping for general surgery residents* “Attendings must recognize and voice the needs of the residents: reach out and confirm their needs and help them make accommodations. Faculty are in position of power to make those changes whereas residents are not.” “I likewise think it is incumbent upon surgical leadership and attending surgeons to verbally set the record straight whenever a breastfeeding resident comes on to service (to tell them that they are not only allowed to take time for breastfeeding, but furthermore that they are EXPECTED to). I think this should all be explicit.” “I think there are certainly plenty of surgical faculty at my institution that still have issues with people taking time to pump…. This fits in the category of surgical faculty at academic institutions thinking someone should always be available based on their own schedule. This is slowly changing, but we have a ways to go in adapting faculty expectations to recognize that there doesn’t always have to be a resident available for everything that they do…”
*Theme 3: A resident leaving an OR case to breast pump can be disruptive, but these disruptions are manageable and acceptable* “Scrubbing out to pump during a case does cause a disruption but no more than if a resident had to use the bathroom urgently. The benefits of supporting residents (and attendings) in breastfeeding far outweigh the minor inconveniences.” “Pumping is inevitably disruptive, speaking as someone who has pumped, but it is still necessary and advisable for most mothers and I support my residents pumping. It's disruptive but we adapt and overcome.” “While it would affect workflow, I don't think those accommodations or adjustments are particularly difficult or detrimental to anyone.” “As with any medical condition, time away from work either for appointments or pumping will affect workflow. I think many pregnant or breastfeeding residents are fully capable of planning ahead and managing. Scrubbing out of a case as a senior or junior resident will definitely affect the case and help will need to be assigned. Planning pumping in between surgery cases or before the case of the day can be safely planned.”
*Theme 4: Communicating breast pumping needs between residents and attendings is key to establishing expectations of all parties involved* “Pumping is a normal behavior and while I don't think the resident should have to announce why they are leaving a case or unavailable for a few minutes, it would be a good idea to share with the attending that they might have to ask for a few minutes to be absent and explain why in a private setting to avoid any misinterpretation of absences. I wouldn't stop a resident from going to the restroom during a case and I don't think many people would have an issue with absence to pump. My observation is that most female residents are excellent time managers and are very unobtrusive about this and I often have no idea when they were doing it.” “Communication about breast pumping to the attending should be strongly encouraged and most attendings will be strongly supportive once they understand that.” “Residents must let their team members know ahead of time when they need the time off for personal time, and that a reasonable explanation can be given so others may understand and stop resentment from building, since these are short but intermittent duration time frames rather than long extended time off.”

### Theme 1: Breast pumping is a natural activity that should be normalized

Surgeons overwhelmingly responded that breast pumping is a normal, healthy activity, and recognized its benefit for both mother and baby. Respondents suggested that increasing education about it, such as its duration and frequency, would be valuable for faculty, helping normalize it.

### Theme 2: Changes are needed to better support breast pumping general surgery residents

Surgeons suggested that changes should be initiated from leadership within residency programs, surgical departments, and institutions, as these individuals have the power to advocate for residents. Additionally, clearly written–out guidelines and policies are needed.

### Theme 3: A resident leaving an OR to breast pump can be disruptive, but these disruptions are manageable and acceptable

A resident leaving a case to breast pump is inevitably inconvenient, but faculty are still supportive of the practice and encourage it. They recognized that if a resident tries to avoid leaving a case, they can be at risk for mastitis and decreased milk supply. Also, it was expressed that many residents already manage this well by pumping before or after cases, causing minimal inconveniences.

### Theme 4: Communicating lactational needs is key to establishing expectations

Respondents stated that misunderstandings could be easily remedied by residents communicating their lactational needs with attendings prior to the start of rotations or cases. This would set expectations, from how long one will be out of the case or if a replacement resident is needed.

## Discussion

Here we report the first study to evaluate the knowledge and perceptions of general surgery teaching faculty on breast pumping in surgical training. Overall, surveyed faculty are largely supportive of breast pumping. However, there appears to be a general lack of knowledge surrounding the practice, which may be hampering greater levels of support amongst faculty. Furthermore, there are different knowledge levels of breast pumping by surgeon age and sex, which is not surprising as commercial at-home breast pumping gained popularity starting in the 1990s [31].

These knowledge gaps warrant increased education for teaching faculty. First and foremost, all faculty should be informed that federal and state laws protect the rights of mothers to have breaks throughout the day to express milk in private locations [32]. This may not be well-known and as a result, comments such as suggesting a resident delay pumping while scrubbed, are ill-informed. Additional education should include information on the average frequency and time required to breast pump, as well as the benefits of pumping [33]. Such education may demystify its practice and increase understanding. While education is important, communication between residents and faculty may be the most valuable intervention [34]. Open communication between all establishes expectations from both sides. Moreover, according to our data, teaching faculty are comfortable discussing such needs and encourage open communication.

Female mentorship is particularly important to female trainees in medicine and surgery [35–38]. As with pregnancy, breast pumping is an opportunity for female faculty with personal experience to provide guidance and support for these residents [39]. Regular check-ins with breast pumping (and all postpartum) residents should be strongly encouraged to assess how they are coping with these major life adjustments. This can include sharing how to balance breast pumping with clinical duties, discussing a residents’ emotional well-being, and ensuring residents are aware of policies and available resources. Female faculty and leadership can be the much-needed advocates for breast pumping residents by ensuring their ability to breast pump and recognizing that there is no one-size-fits-all experience for pumping.

Furthermore, respondents expressed the need for policies that clearly establish support for breast pumping. These responses reflect recent efforts across specialties to increase inclusivity by providing private and accessible lactation facilities and cultivating cultural change [40–46]. Lactation facilities should be private and easily accessible to the operating room for surgical residents (and attendings). They should include a sink and refrigerator for cleaning parts and storage of breast milk. Although residents should not be expected to continue working while pumping, providing computers with access to the electronic medical record may minimize potential workflow disruptions if a resident wishes and is able to use it simultaneously while pumping.

Additionally, to further support residents breastfeeding and breast pumping, it is important to promote policy change for maternity leave, as the duration of breastfeeding has been shown to be positively associated with the duration of maternity leave [6, 47]. Despite recent changes by the American Board of Surgery, some advocate further changes and flexibility to the current leave policy to better support residents with children [48–50]. Increasing the duration of maternity leave may further support and encourage breastfeeding amongst surgical residents.

Though our study elucidates a unique perspective on breast pumping, we do acknowledge its limitations that restrict the generalizability of our results beyond those surveyed. Biases, including response, sampling, recall, and social desirability, cannot be overlooked. For example, individuals more passionate about the topic may have been more likely to respond, potentially skewing the results to a more favorable view of breast pumping. Our small sample size also likely does not represent the current general surgeon workforce or general surgery residency program demographics, thus, under/over-representing certain groups such as female surgeons and academic programs [51, 52]. Despite these limitations, we do believe our results are the first of their kind and notably contribute to conversations and efforts to improve the surgical training environment for residents, and warrant dissemination within the surgical community.

As we know from existing literature, as well as anecdotal and personal experience, residents experience conflicting feelings about stepping away from their clinical duties to provide breastmilk for their child [20]. Our results demonstrate that at least of those surveyed, faculty are supportive of breast pumping, and thus, residents should feel empowered to engage in a healthy activity performed by millions of women worldwide. Although our results appear favorable towards breast pumping, we are cautious to interpret these sentiments as universal amongst all surgical faculty and programs. We, therefore, take this opportunity to challenge all those in the surgical community to critically assess the culture and environment surrounding breast pumping at your institutions and programs. As we have outlined above, there are ample opportunities for areas of improvement, and with such evaluation, individual programs can better understand where they fall on the spectrum of support and how they can better support their breast pumping residents. By improving support for breast pumping residents, we may take the necessary strides towards a more inclusive culture for women in surgery.

## Supplementary Information

Below is the link to the electronic supplementary material.

Supplementary file1 (DOCX 18 KB)

## Declarations

### Previous Presentations

An abstract of this manuscript was presented as an Oral Presentation at the 18th Annual Academic Surgical Congress in Houston, Texas, USA on February 7–9, 2023.
