# Supplementary material for: General Surgery Faculty Knowledge and Perceptions of Breast Pumping Amongst Postpartum Surgical Residents
Source: World J Surg. 2023 Apr 27;47(9):2092–100. doi: 10.1007/s00268-023-07005-5 (PMC10387458; doi:10.1007/s00268-023-07005-5)
Supplement: Supplementary file 1 — Supplementary file1 (DOCX 18 KB) [file 268_2023_7005_MOESM1_ESM.docx]

**Supplemental Material: Breast Pumping Perceptions Survey**

Are you a surgical attending who works with general surgery residents on a regular basis (i.e. at least weekly)? *REQUIRED* *Answer of “No” exits the survey.”

- Yes
- No

**Section 1: Demographics**

Please answer the following questions about yourself and your surgical practice.

1. What is your sex?
   1. Male
   2. Female
   3. Other
2. How old are you?
   1. <30 years old
   2. 30-39 years old
   3. 40-49 years old
   4. 50-59 years old
   5. 60-69 years old
   6. ≥70 years old
3. What is your race? Mark ALL that apply.
   1. White or Caucasian
   2. Black or African American
   3. American Indian or Alaska Native
   4. Asian
   5. Native Hawaiian
   6. Other Pacific Islander
   7. Other
4. What is your ethnicity?
   1. Non-Hispanic
   2. Hispanic
5. How long have you been practicing as an attending surgeon?
   1. 0-5 years
   2. 6-10 years
   3. 11-15 years
   4. 16-20 years
   5. 21-25 years
   6. >25 years
6. What is your primary surgical specialty?
   1. Acute Care/Trauma/Critical Care
   2. Bariatric/Metabolic
   3. Breast
   4. Burn
   5. Cardiothoracic
   6. Colorectal
   7. Endocrine
   8. General
   9. Hepatopancreaticobiliary
   10. Pediatric
   11. Plastics
   12. Surgical Oncology
   13. Transplant
   14. Vascular
   15. Other: ____
7. In what region of the United States do you practice?
   1. Northeast (CT, MA, ME, NH, NJ, NY, PA, RI, VT)
   2. Southeast (AL, AR, DC, DE, FL, GA, KY, LA, MD, MS, NC, PR, SC, TN, VA, WV)
   3. Midwest (IA, IL, IN, KS, MI, MO, MN, ND, NE, OH, SD, WI)
   4. Southwest (AZ, NM, OK, TX)
   5. West (AK, CA, CO, HI, ID, MT, NV, OR, UT, WA, WY)
8. Describe the location of the practice you work in.
   1. Rural
   2. Urban
   3. Suburban
9. Describe the type of hospital you practice in.
   1. Community
   2. Academic
   3. Hybrid
   4. Federal
   5. Other
10. Are you involved in the leadership of your general surgery residency program (e.g., Program Director, Associate Program Director, etc.)?
    1. Yes
    2. No
11. Do you have biologic children?
    1. Yes
    2. No
       1. Skip questions 12, 13, 14
12. When in your career did you or your partner have your children? Mark ALL that apply.
    1. Before medical school
    2. During medical school
    3. During residency
    4. During fellowship
    5. As an attending
13. Did you breast pump as a resident, fellow, or attending? *Software logic will generate this question for appropriate respondents based on previous answers.*
    1. Yes
    2. No
14. Did your partner breast pump? *Software logic will generate this question for appropriate respondents based on previous answers.*
    1. Yes
    2. No

**Section 2: Introductory Questions**

The following questions refer to ALL general surgery residents. Please read each statement and answer accordingly.

1. Female general surgery residents perform equally as well as male general surgery residents.
   1. Strongly Disagree
   2. Agree
   3. Neither Agree nor Disagree
   4. Disagree
   5. Strongly Agree
2. I would allow a surgery resident, male or female, to break scrub from a case without asking probing questions as to why.
   1. Strongly Disagree
   2. Agree
   3. Neither Agree nor Disagree
   4. Disagree
   5. Strongly Agree

**Section 3: Breast Pumping**

The following questions will ask you about your perceptions of breast pumping in general surgery residents. Please read each question or statement and answer accordingly.

1. The nearest operating room accessible lactation facilities available at my hospital for residents to use are found…
   1. Adjacent to the ORs
   2. On the same floor as the ORs
   3. In the same building, but different floor as the ORs
   4. In a different building than the ORs
   5. There are no lactation facilities
   6. I don’t know
2. I would feel comfortable having a general surgery resident discuss their lactation needs with me, such as telling me that they need to scrub out of a case to pump.
   1. Strongly Disagree
   2. Agree
   3. Neither Agree nor Disagree
   4. Disagree
   5. Strongly Agree
3. There is adequate time during the work day for a general surgery resident to pump.
   1. Strongly Disagree
   2. Agree
   3. Neither Agree nor Disagree
   4. Disagree
   5. Strongly Agree
4. I would support a general surgery resident if they wished to visit the on-site childcare facility to breastfeed their child instead of pumping.
   1. Strongly Disagree
   2. Agree
   3. Neither Agree nor Disagree
   4. Disagree
   5. Strongly Agree
5. How long do you believe a single breast pumping session should take (i.e. the time needed to leave the OR, go to a lactation room, pump, clean up, and return to the OR?)?
   1. 0-15 minutes
   2. 16-30 minutes
   3. 31-45 minutes
   4. 46-60 minutes
   5. >1 hour
   6. I don’t know
6. How often, on average, do you think a female general surgery resident would need to pump during the work day?
   1. Every 1-2 hours
   2. Every 3-4 hours
   3. Every 5-6 hours
   4. Every 7-8 hours
   5. I don’t know
7. The workflow of the operating room is not impacted when a general surgery resident needs to break scrub to pump.
   1. Strongly Disagree
   2. Agree
   3. Neither Agree nor Disagree
   4. Disagree
   5. Strongly Agree
8. I expect a general surgery resident to find another resident to scrub into a case when they scrub out to pump.
   1. Strongly Disagree
   2. Agree
   3. Neither Agree nor Disagree
   4. Disagree
   5. Strongly Agree
9. A general surgery resident should continue working while pumping in a lactation room, such as by charting on the computer.
   1. Strongly Disagree
   2. Agree
   3. Neither Agree nor Disagree
   4. Disagree
   5. Strongly Agree
10. Team workflow and patient care are not impacted by a general surgery resident taking time to pump during the workday.
    1. Strongly Disagree
    2. Agree
    3. Neither Agree nor Disagree
    4. Disagree
    5. Strongly Agree
11. I support a general surgery resident’s need to pump.
    1. Strongly Disagree
    2. Agree
    3. Neither Agree nor Disagree
    4. Disagree
    5. Strongly Agree
12. My institution harbors a supportive culture and environment towards the lactation needs of general surgery residents.
    1. Strongly Disagree
    2. Agree
    3. Neither Agree nor Disagree
    4. Disagree
    5. Strongly Agree
13. Please provide any comments that you feel are important to share about perceptions of breast pumping general surgery residents. ____
